# Systematic Exploration of Small-Molecule Binding via a Large Language Model Trained on Textualized Protein–Ligand Interactions

**DOI:** 10.3390/molecules30234516

**Published:** 2025-11-22

**Authors:** Taeseob Lee, Heehoon Jung, Ahnjae Jung, JaeWoong Min, Jong Hui Hong, Bin Claire Zhang, Jongsun Jung

**Affiliations:** 1 Syntekabio Inc., 18 Gukjegwahak 17-ro, Yuseong-gu, Daejeon 34002, Republic of Korea; 2 Department of Digital Health, Samsung Advanced Institute for Health Sciences & Technology, Sungkyunkwan University, Seoul 06355, Republic of Korea; 3 Department of Artificial Intelligence, Korea University, Seoul 02841, Republic of Korea; 4 Cerebras Systems Inc., 1237 E. Arques Ave, Sunnyvale, CA 94085, USA; 5 Syntekabio USA Inc., 425 Fifth Ave. Suite 505, New York, NY 10016, USA

**Keywords:** textualized binding interaction, GPT application, categorized chemical properties, AI drug discovery

## Abstract

Emergent Large Language Models (LLMs) show impressive capabilities in performing a wide range of tasks. These models can be harnessed for biophysical use as well. The main challenge in this endeavor lies in transforming 3D chemical data into 1D language-like data. We developed a method to transform molecular data into language-like data and tokenize it for LLM use in a biophysical context. We then trained a model and validated it with a known protein–ligand complex. Using the pre-trained result, the model can assess the chemical properties of targets, detect shared binding properties and structures, and reveal related drugs. The model and the synthetic language to describe binding interactions uncovered novel protein–protein networks influenced by ligands, indicating functionally related yet previously unreported interactions.

## 1. Introduction

The investigation into forecasting binding interactions between ligands and proteins has been instrumental in revealing novel therapeutic target proteins and identifying potential pharmaceutical agents [1]. To address these complexities inherent to chemical binding interactions, the use of artificial intelligence (AI) combined with three-dimensional (3D) virtual screening (VS) has become widespread and shows promising potential as a viable approach [2]. Biophysical data depict spatially and temporally complex systems with intricate interactions, thus requiring specialized approaches, data structures, and computational methods such as deep learning for processing and analysis.

A central challenge in the computational elucidation of protein–ligand interactions lies in developing computationally tractable representations for molecular structures. Advanced and recent AI models, such as AlphaFold3 [3] and Boltz-2 [4], exemplify a new paradigm by training directly on 3D raw atomic coordinates. In contrast, models based on the AlphaFold2 architecture, like OpenFold [5], are trained primarily on 1D protein sequences and 2D multiple-sequence alignments (MSAs) to infer structural information. This architectural distinction is critical: OpenFold focuses on structural prediction for proteins by determining residue-specific reference frames and torsion angles. This protein-centric representation is well-suited for predicting protein–protein interactions but is fundamentally ill-equipped to model the diverse chemical space of small-molecule ligands. Conversely, AlphaFold3 and Boltz-2 overcome this limitation. AlphaFold3 employs a diffusion module to generatively predict the 3D atomic coordinates for the entire biomolecular complex simultaneously. Boltz-2 utilizes a similar denoising module to generate the 3D structure but further extends this capability by incorporating a dedicated affinity module, which explicitly calculates binding strength to provide a quantitative measure of interaction potency. While these models have achieved formidable success, significant performance gaps remain in capturing accurate physical interaction behaviors [6,7]. This limitation may be a direct consequence of their training paradigm: the models are primarily supervised on static, end-state structures rather than the binding interactions themselves, potentially because the dynamic, biophysical process of binding is not yet well-represented in a computationally trainable format.

A few techniques have been developed to address this challenge, including transforming SMILES (the simplified molecular input line entry system) [8] format into 2D or 3D data structures [9]. More recently, AI language models are used to better dissect these string-based data [10]. Large Language Models (LLMs), an emerging deep-learning method, show impressive capabilities in performing a multitude of tasks of different natures [11,12,13], promising to overcome this challenge. Many of these tasks focus on common language uses such as summarization, editing, and translation [14]. These models can also be harnessed to perform less conventional tasks, including analyzing molecular structures and chemical features, especially in the context of protein–ligand interactions, protein–protein interactions, biophysical network analysis, protein design, and drug discovery [15,16,17].

A critical step in applying LLMs to biophysical data is transforming complex, 3D spatial data into language-like, 1D string-based data that can be further processed by LLMs. The challenge in this transformation lies in preserving information beyond atom identity, chemical bonds, and electrochemical charges. Deep learning models such as 3D-convolutional neural networks (3D-CNNs) and spatial graph convolutional neural networks (SG-CNNs) [18] can use graph networks to represent compounds; however, this representation does not necessarily encompass information related to electrochemical interactions or medium-dependent phenomena [19,20,21].

We introduce a novel approach for integrating 3D binding interactions data into LLMs, addressing a central challenge in transforming biological data into a “machine-readable” format, especially by LLMs. Our approach defines a new synthetic, “machine-readable” language that can represent molecular, 3D spatial data in a fashion that encapsulates biochemical features. We trained a Generative Pre-trained Transformer (GPT) model, a model originally developed by OpenAI [11], to analyze and predict text-converted 3D binding simulation data. The model, named “3D binding mode GPT” (3bmGPT), demonstrates that our method can effectively represent biochemical data in a format amenable to analysis by LLMs, both by reproducing previously validated results and by uncovering new features to known biophysical interactions.

## 2. Materials and Methods

### 2.1. Text Conversion for 3D Binding Prediction

**Figure 1:**
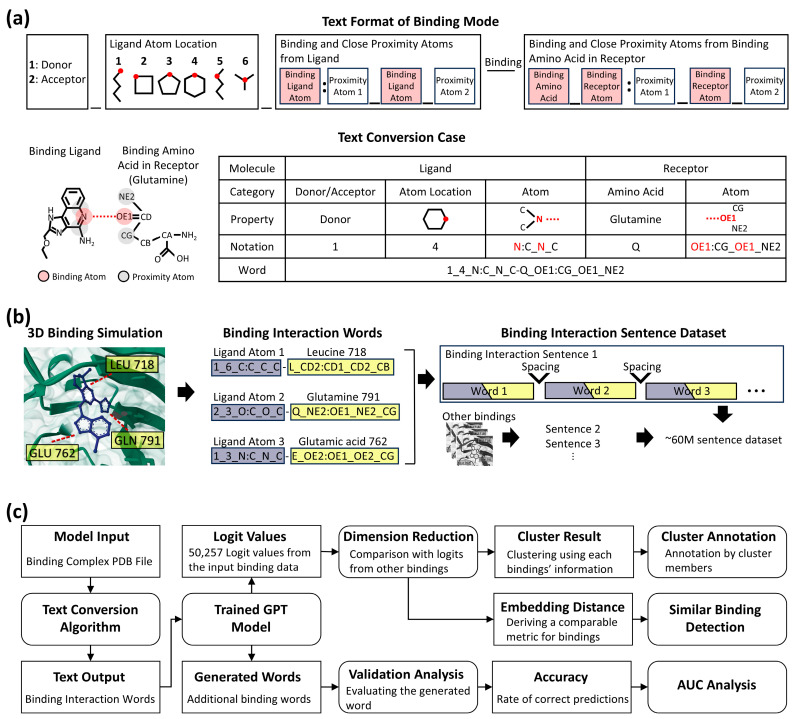
Overview of the text conversion, dataset generation pipeline, and model application framework. (**a**) Detailed format of text conversion from binding mode and sample case. The upper part (Text Format of Binding Mode) shows the structure of a word representing a single binding interaction. The lower part (Text Conversion Case) depicts the conversion of the binding part in a ligand and the binding amino acid in a receptor. The table from the text conversion case illustrates how binding properties change by given notation, resulting in a binding interaction word. (**b**) Illustration of dataset generation for binding interaction sentences. The first step simulates binding interactions between a small molecule (blue) and amino acids (yellow). The second step visualizes their conversion into binding interaction words (ligand in purple, receptor in yellow). The final step illustrates their transformation into sentences, generating approximately 60 million sentences from multiple simulations. Ellipses denote continuous words and sentences. (**c**) Flowchart detailing the model, analysis steps, and input/output data. Square boxes indicate input/output data, either initially introduced or generated by a process. Round-edged square boxes represent a process, such as a programmed model or analysis step, that takes input data and generates output data. Lines with arrows illustrate the flow of data and the sequence of operations.

We developed a synthetic language based on the Dictionary of Secondary Structure in Proteins (DSSP) algorithm [22] to represent binding interactions data. The interaction of binding residues in the binding pocket has a unique fragment composed of three atoms, and one specific atom of each fragment was defined as a donor or acceptor (Figure 1a). From these atoms, a bond forms between an atom in a ligand and a target protein. This bond formation constitutes a “binding interaction word.” Subsequently, each discrete interaction, whether it is a simple contact or one of several interactions originating from a single atom, is individually represented as a word. The complete aggregation of all such words then constitutes the comprehensive binding interaction, forming a “binding interaction sentence” (Figure 1b). Because each word depicts a single protein residue and ligand state at a specific time point (i.e., a specific ligand in a specific spatial orientation), changes in this state, such as shifts in the spatial orientation that occur over time, will yield different words and new binding interaction sentences.

To encapsulate these biochemical dynamics, we collected data from the 3D interaction binding dataset, CrossDocked2020 [23], which has 18,450 protein–ligand complexes with 22.6 million interaction poses specified by Pocketome [24], as input for our method. We then simulated data using a CNN affinity algorithm [25] to find more interaction poses, generating 120 additional poses per complex to account for binding flexibility, such as side chain variations. The interaction of binding residues in the pocket had a unique fragment composed of three atoms, and a specific atom of each fragment was defined as a donor and an acceptor. The ligand also contained donors and acceptors according to the same principle. As a rule, the donor of a residue interacts with the acceptor of a ligand, and the acceptor of a residue interacts with the donor of a ligand. Therefore, if the combination of the donor–acceptor of a residue–ligand pair was defined as a word, and all other ligand fragments interacting with a specific residue were defined as sentences. These sentences could be created with slightly different characteristics, either when new ligands were used as input or when new sentences were created by each motion of an existing ligand. This conversion from 3D information to 1D, text format, generated approximately 60 million trainable paragraph data by the language AI model.

We trained an LLM on the text-converted data to comprehensively analyze the nature of binding interactions and to evaluate the potential of this approach for studying protein–ligand interactions. A schematic overview of the model’s architecture and its applications is illustrated in Figure 1c, which delineates the input/output data formats and the analysis processes for different objectives. The detailed methodology is described in the following sub-sections, and the corresponding results and their interpretation are discussed in Section 3 (Results).

### 2.2. Validation of Text Conversion Using Tight-Binding Designed Data

To simplify molecular dynamics and ensure accurate predictions, we used 463 simulated binding datasets with tight-binding ligands for 16 targets: BACE, CDK2, CDK8, CMET, EG5, HIF2A, JNK1, MCL1, P38, PFKFB3, PTP1B, SHP2, SYK, THROMBIN, TNKS2, and TYK2 [26]. Ligands were extracted from the Protein Data Bank (PDB) [27] data to SMILES sequences; then, similarities were computed using the Tanimoto coefficient (TC) [28]. TCs were calculated using the interaction fingerprint method [28]. Firstly, SMILES sequences were parsed to generate fingerprint format by the rcdk package version 3.8.1 [29]. The distance between fingerprints was calculated by the fingerprint package version 3.5.7. A heatmap was drawn by the NMF package version 0.27 [30]. Shared words were defined as those containing the same elements, including adjacent elements, and the percentage of shared words was calculated as the number of shared words divided by the total query word set. Statistical significance was assessed using a *t*-test in R (version 4.2.2).

$$Shared\;Word\;Percentage\;\left(\%\right)=\frac{\left|Word_{A}\cap Word_{B}\right|}{\left|Word_{A}\cup Word_{B}\right|}\times 100$$

### 2.3. Data Preparation with Tokenization

As a data filtration step, we excluded binding sentences with five or fewer words. This threshold was based on the observation that insignificant or non-specific interactions typically generate a correspondingly low number of binding words. Then, the protein–ligand dataset was partitioned into training, validation, and test sets in a 7:2:1 ratio. Each paragraph was a list of words represented in strings. We adopted two tokenization methods, and each method included special tokens [PAD], [SEP], and [UNK] in the vocabulary. First, we used word-based tokenization by treating each word as a token. All unique tokens in the entire dataset, plus the special tokens, constituted the vocabulary, which had a size of 143,018. Second, we trained a Byte-Pair Encoding (BPE) tokenizer on the dataset text, where all words within a paragraph were concatenated by a space character and paragraphs were separated by a newline character. The total vocabulary size was set to be 50,257. One potential benefit of BPE tokenization, which needs to be further examined, is the ability to keep the vocabulary size contained even when additional new words are added to the protein–ligand dataset. After the BPE tokenization, space and newline characters were taken out of the training tokens to avoid the excessive appearance of these two characters. For both tokenization schemes, tokens from the same paragraph constituted one sample. For all models trained, the maximum sequence length was set to 1071, equivalent to the number of tokens in the longest sample. Shorter samples were padded with the [PAD] token to the maximum sequence length. Training was performed with one epoch of the training tokens. To verify the reproducibility of our results, the whole process was performed an additional time.

### 2.4. Training Data for GPT and BERT LLMs

We used the protein–ligand token data to train GPT-2 [11]. Two GPT-2 backbone sizes were trained in PyTorch (version 2.2.1). GPT-2 small had a hidden size of 768, 12 hidden layers, and 12 attention heads and was trained with a batch size of 112, AdamW optimizer, a 6e-04 peak learning rate, 1600 warm-up steps, and a linear learning rate scheduler. GPT-2 medium had a hidden size of 1024, 24 hidden layers, and 16 attention heads and was trained with a batch size of 100, AdamW optimizer, and a 2e-04 peak learning rate. Then, we ran the studies on the Cerebras Wafer Scale Cluster with weight streaming technology [31] and software release 1.8.0. Cerebras uses the Wafer Scale Engine (WSE), which has 850,000 cores with 40 GB of on-chip memory. Training times are shown in Table 1.

The BERT (Bidirectional Encoder Representations from Transformers) model [13] was trained on the same dataset using PyTorch (version 2.2.1). Similarly to the GPT model, it was configured with a hidden size of 768, 12 hidden layers, and 12 attention heads. Training was performed using three NVIDIA Tesla V100 GPUs with multithreaded processing.

### 2.5. Deriving Binding Words and Logits Using the Trained LLM

The trained model was archived as a set of files comprising model weights, configuration, and tokenizer data. As the model was trained on binding sentences described in Section 2.1, the input data must also be binding sentences generated from query PDB files. These query PDBs must contain a binding complex with a target protein and ligand to facilitate the analysis of further bindings or related binding properties. The PDBs were preprocessed for trimming and standardization, then subjected to binding detection and binding text generation. The resulting text can be directly applied to the loaded model to generate subsequent binding sentences or to calculate logits that quantify binding properties. To interpret the results, users can directly examine the generated binding sentences or compare the logit values against a reference dataset (e.g., the provided 10k dataset).

### 2.6. Validation of LLMs Using Practical Binding Data

To validate word generation and compare LLM performance, we used binding data from the drug Rebastinib and its target. ABL1. This target was chosen because its key bindings have been reported, and its binding is not highly unique, as it interacts with multiple proteins in the same kinase family [32]. This allowed us to assess various predictive capabilities of LLMs. From its PDB data, 30 binding interaction words were generated, and the first 10 words were input into the pre-trained GPT and BERT models to predict the next binding interaction word using the same parameters: temperature 0.7 and repetition penalty 2. This process was repeated 1000 times for each model, and the total results were analyzed for performance comparison.

Generated words were categorized into four distinct groups: (1) a word was labeled “query adjacent” if it exactly matched the next word in the input sequence (e.g., the 11th word in the Rebastinib case); (2) it was labeled “exists in original” if the word was not the correct adjacent word but was present elsewhere in the original words (e.g., the model predicts the 15th word or 3rd word instead of the 11th); (3) it was considered a “variation of existing words” if it shared binding elements with an original word but was not an exact match (e.g., the generated word is “1_6_C:N_C_C-E_O:C_N_CA,” while the 16th word is “1_4_C:N_C_C-E_O:C_N_CA”); and (4) it was classified as “completely new” if it did not fall into any of these categories. Prediction accuracy was assessed using logit values to calculate AUC scores, with separate measurements: word (1) was considered as the only true positive, and words (2) and (3) were combined as true positives. The first measurement indicated how accurately the model predicted the next word, while the second reflected its ability to generate related bindings.

We also generated words from binding simulation data between EGFR and ligand AEE788, a dual-targeting inhibitor for both EGFR and ERBB2 [33]. A total of 23 binding interaction words were generated from the simulation, and the first 7 words were input into the GPT model to generate 12 additional words with default parameters. These 12 words were then searched in the previously generated word dataset to identify shared words with other targets, and protein sequence similarities were calculated using BLAST [34] (version 2.16).

### 2.7. Cluster Analysis

To compare prediction results for target proteins, we randomly selected 10,000 sentences and applied them to GPT model, which was pre-trained using PyTorch (version 2.2.1) based on the modified GPT-2 medium configuration (See Section 2.4). The average logit values for each token were calculated, producing a 10,000 × 50,257 matrix. Because the matrix was of high order, we applied feature selection and dimensionality reduction techniques using the Seurat package version 4.2.1 [35] in R version 4.2.2. High standard deviation token vectors were selected to reduce the number of parameters to 737, followed by principal component analysis (PCA) for further dimension reduction. Utilizing 100 principal components, UMAP analysis with Louvain clustering was conducted to group target proteins with similar characteristics; then, we identified token vectors with distinct values for each cluster, ordered by the false discovery rate (FDR) value. We repeated our analysis 3 times (hereby noted as “trials”), all yielding consistent results, thus ascertaining the analysis reproducibility (Supplementary Figure S4).

### 2.8. Analysis for Biological Function

Each sentence’s data was annotated based on its target protein, with each data point representing a distinct target. Data points associated with the same target protein were color-coded on the UMAP plot to visualize the distribution of these target proteins. Subsequently, we examined the proportions of clusters corresponding to each target protein. To discern the biological functions, we converted the names of target proteins into gene names using UniProt ID mapping [36]. Genes related to tyrosine kinase (TK) and serine/threonine kinase (STK) were retrieved from Gene Ontology [37] for further analysis of kinase-related binding interactions. Cluster characteristics were determined by applying gene members within each cluster to GO enrichment analysis for biological processes [38]. Function terms that appeared in more than 10 clusters were filtered out. From the remaining terms, we selected the function term with the lowest FDR value within a cluster to represent the biological function of that cluster. Additionally, we computed the average vector values for each cluster and used hierarchical clustering to compare these values. This hierarchical ordering allowed us to group the clusters into six categories, revealing common biological function terms identified through GO analysis that described the larger cluster’s characteristics. Furthermore, we identified genes related to insulin, sex hormones, and vitamin receptors from Gene Ontology to investigate the distribution of their binding interaction sentences.

### 2.9. Analysis from Applied Interaction Data for Biological Function and Ligand Similarity

First, we used the model to generate binding interaction words and logit values from the PDB-formatted data. These newly generated logit values were then combined with the 10,000 pre-analyzed reference data to generate new UMAP coordinates. Subsequently, we marked the location of the newly introduced data on the UMAP plot and generated a radar plot depicting the normalized distances between the new data’s location and the centroids of each cluster, annotated by biological function. To quantify structural similarity, Tanimoto coefficients (TCs) were calculated between the ligand extracted from the input PDB complex and each of the 10,000 ligands derived from the reference datasets, yielding 10,000 corresponding similarity scores. To visualize the TC trend across the UMAP space, the coordinates were first partitioned into 100 segments (bins). The average TC within each bin was then calculated and rendered as a line plot, revealing the relationship between UMAP proximity and structural similarity. Finally, the 20 nearest interaction data points to the input data were selected based on UMAP coordinates for network analysis. For the network analysis, connections were drawn exclusively for the 10 pairs with the highest TC values among all combinations. The ligands from the 20 nearest interactions were queried against the drugbank database [39], which identified several known drugs with potential for repurposing. Subsequently, the structures of these known drugs were compared with that of the input ligand to identify shared molecular features.

### 2.10. Benchmarking Analysis

The target identification performance of 3bmGPT was benchmarked against ProBiS [40] and Foldseek [41] using a test set of 23 randomly selected PDBs. For ProBis and Foldseek, predictions were generated via their web servers. For 3bmGPT, PDBs were converted to text to generate logit values. The target was then assigned based on the most common protein among the 100 nearest neighbors, as determined by Euclidean distance in the logit space. To further assess the model’s capacity to discriminate between structurally similar targets, we used a set of 9 kinases from the KLIFS [42] dataset. The 3bmGPT logit distance, ProBiS E-value, and Foldseek Z-score were calculated for this set and used to determine the AUC, allowing for a direct comparison of their discriminative power.

## 3. Results

### 3.1. Synthetic Language to Represent 3D Protein–Ligand Binding Data and Evaluation

**Figure 2:**
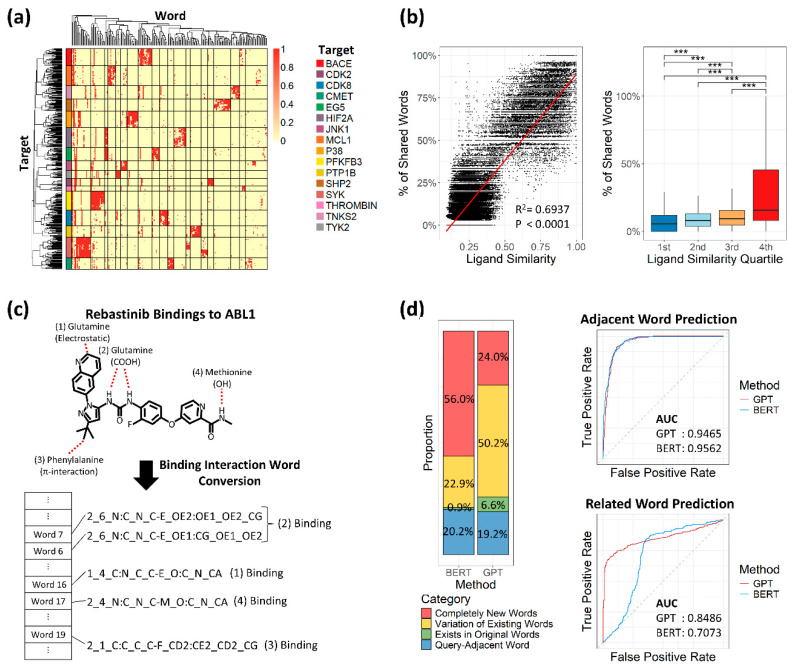
Validation of text conversion and large language model performance. (**a**) Heatmap of 463 tightly designed bindings across 16 target proteins. Rows represent individual binding samples, ordered by hierarchical clustering and annotated with colors corresponding to each target protein. Columns are ordered by hierarchical clustering of textualized binding interactions. Distinct clusters are separated by lines. (**b**) Relationship between ligand similarity and percentage of shared binding interaction words. Left: The dot plot shows the correlation between ligand similarity (*x*-axis, Tanimoto coefficient) and the percentage of shared binding interaction words (*y*-axis). A linear regression line is shown in red, with R^2^ and *p*-value indicated. Right: Box plots display the distribution of shared word percentages across quartile groups; 1st to 4th represent each quartile range. Statistical significance between groups was measured by a *t*-test: *** *p* ≤ 0.001. (**c**) Example of key binding capture (Rebastinib). The red dashed lines indicate bonds between the ligand and protein atoms. (**d**) Comparison of GPT and BERT based on generated word proportions and area under the curve (AUC). Left: Bar plots show the proportion of words generated by each model. Right: AUC plots for adjacent and related word prediction, with GPT in red and BERT in blue.

Validation of the text conversion methodology was performed using a curated benchmark set of 463 datasets [26]. These datasets were intentionally selected because they represent static and high-affinity interactions, providing a clear test of our model’s ability to capture fundamental binding properties. Although the ligands within this set were structurally distinct—reflecting different molecular design approaches for the same target—the textual representations generated by our model showed remarkable convergence. As a result, the shared binding words were distinctive enough to allow for accurate clustering of these datasets based on their target proteins (Figure 2a). Moreover, ligand similarity showed a moderate linear correlation with the percentage of shared words (R2: 0.6937), and all structurally distant ligand groups exhibited statistically significant differences (Figure 2a). To further explore the potential of binding interaction words, we applied this binding data to LLMs to gain deeper insight into binding dynamics.

To test text-converted bindings and select a suitable LLM, we first converted binding data between the drug Rebastinib and its target protein ABL1, which involves four key bindings [32]. These key bindings were accurately detected and represented as binding interaction words, regardless of binding type (Figure 2c). We then applied partial words from the Rebastinib–ABL1 bindings to two LLMs, GPT and BERT, both trained on our 60 million binding interaction sentences, to assess prediction performance. With identical parameters, BERT and GPT generated similar proportions of adjacent words (20.2% and 19.2%, respectively), but BERT generated more unrelated new words (Figure 2d). When considering true predictions as adjacent words, both models showed similar performance based on AUC values. However, when true predictions were defined as words related to query words, GPT outperformed BERT with an AUC of 0.8486 versus 0.7073 (Figure 2d). Based on this result, we focused on developing the GPT model.

We then used the epidermal growth factor receptor (EGFR), a well-studied target protein renowned for its substantial implications in tumor growth and metastasis [43], to evaluate the model’s application in word generation. From its simulation with ligand AEE788, we generated 12 words, and four of these words corresponded to interaction words observed in complexes of other protein targets: ABL1, VGFR2, EPHA2, and CDK2 (Supplementary Figure S1a). Notably, all these targets, except for CDK2, are well-known targets for tyrosine kinases (TKs), the same family to which EGFR also belongs [44,45]. CDK2, a well-established target for serine/threonine kinase (STK) [46], was also identified as sharing residues with EGFR. Both TK and STK are known to possess similar structural traits [47]. Within our trained dataset, the target proteins associated with TK and STK exhibited no significant differences in sequences, whereas their distinction from other proteins demonstrated statistical significance (Supplementary Figure S1b). Thus, the GPT model aptly captured this structural resemblance, pointing to key binding interactions that can provide valuable insights into the relationship between EGFR and the other related target proteins.

### 3.2. The Model Preserves the Characteristics of Coexisting Binding Nature

**Figure 3:**
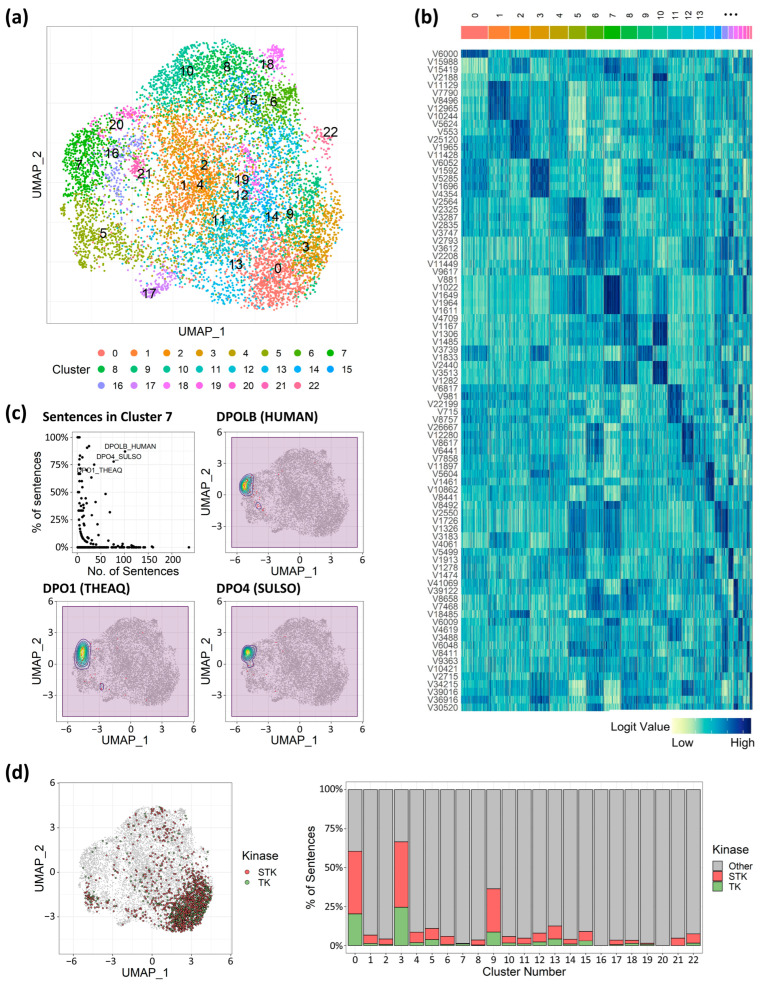
Target protein characteristics unveiled via cluster analysis of token vectors. (**a**) UMAP analysis with 23 clusters. (**b**) Token vector values heatmap. Dark-blue color indicates a higher value of vector values, and light-green color indicates a lower value. (**c**) Dot plot for proportion and number of sentences in cluster 7, and density plot on UMAP coordinates for sentences targeting DNA polymerase. (**d**) Plotting sentences for serine/threonine kinase (STK) and tyrosine kinase (TK), and the proportion of sentences for the kinases. STK in red, TK in green.

In natural language processing (NLP) tasks, results can be classified to identify aspects like the emotional tone of a text. This is often achieved not by focusing on the single most likely next token, but by treating the entire logit distribution as a feature vector that represents the input’s overall properties. In our model, protein–ligand interactions were predicted based on binding interaction words, utilizing the logit values corresponding to each tokenized vector generated by the NLP model. This approach enabled the application of NLP clustering techniques to identify trends in binding natures. We clustered 10,000 randomly selected binding representations into 23 clusters (Figure 3a) and observed that binding complexes tended to aggregate based on their respective target proteins (Supplementary Figure S2). The 23 unsupervised clusters also grouped sentences targeting the same proteins into coherent clusters. For example, cluster 2 and cluster 5 contained approximately 88% and 85% of the sentences generated from mouse ACES and horse ADH1E, respectively (Supplementary Table S1). Similarly, sentences generated from proteins encoded by analogous genes were closely located, suggesting a higher likelihood of clustering together.

Of note, binding interaction sentences generated from the DNA polymerase protein family demonstrated that shared structural or binding attributes transcended factors such as species and potential data biases in shaping probabilities. Within cluster 7, over 75% of sentences related to DNA polymerase genes—including DNA polymerase beta from humans (denoted as DPOLB HUMAN), DNA polymerase 1 from Thermus aquaticus (denoted as DPO1 THEAQ), and DNA polymerase IV from Sulfolobus tengchongensis (denoted as DPO4 SULSO)—were congregated (Figure 3c), and their corresponding vectors (881st, 1022nd, 1649th, 1964th, and 1611th) values were significantly higher compared to other clusters (Figure 3b). This suggested that these specific vectors could be pivotal in representing distinct binding properties unique to DNA polymerase, setting them apart from other target-binding complexes. Instead of originating from the same genes, the target proteins involved in the kinases, TK and STK, held particular significance due to their substantial implication in cancer therapy [48] and the similarities in their binding interactions. Intriguingly, the involved sentences appeared to intermingle predominantly within clusters 0 and 3 (Figure 3d), suggesting that the binding interactions indeed possessed striking similarities, accurately captured by the 3bmGPT. However, while TK and STK were not isolated into distinct clusters, the separation of clusters 0 and 3 indicated the potential existence of finer-grained characteristics that could serve to distinguish between them (Figure 3b). This pattern of distinct vector values was similarly evident across the remaining clusters associated with each target protein, potentially serving to uncover cofactors and distinguishing elements essential for the binding interactions of diverse target proteins.

### 3.3. The 3bmGPT Model Clustered Binding Interactions from Associated Molecular Functions by Distinct Features

**Figure 4:**
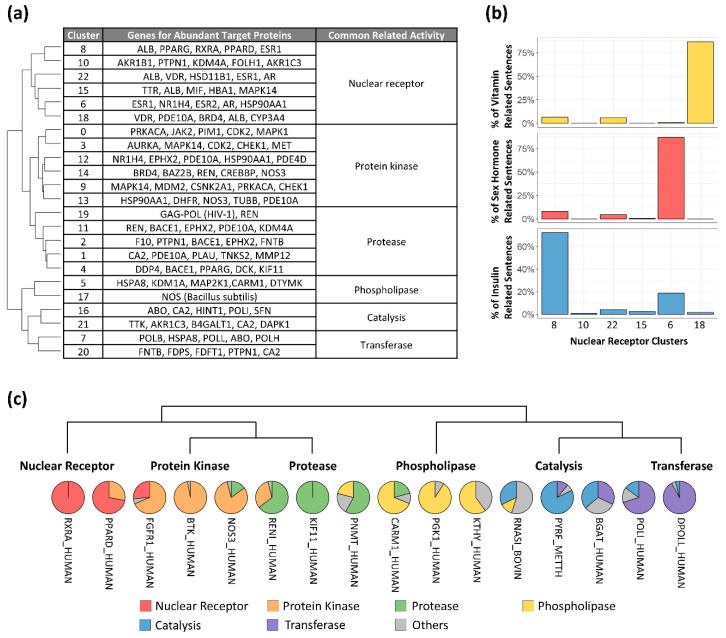
Functional group for clusters and multifunctional target proteins. (**a**) Hierarchically ordered clusters with biological functions. Cluster numbers are the same as in Figure 1a. (**b**) Percentage of binding interaction sentences related to specific functions within the nuclear receptor clusters: vitamin (yellow), sex hormone (red), and insulin (blue). Cluster order is hierarchical. (**c**) Target proteins across functional groups ordered by the nearest functional group in hierarchical clustering.

Diverse biological functions have been reported to exhibit a robust correlation with distinct types of proteins [49]. Particularly, ligand binding sites within protein complexes have a substantial impact on their functions [50]. Given these factors, our cluster results also revealed distinct biological and molecular functions within each trained dataset. Employing Gene Ontology annotations [38], we identified common molecular functions within each cluster group, resulting in six functionally similar categories, including nuclear receptor, protein kinase, protease, phospholipase, catalysis, and transferase functions (Figure 4a). Importantly, the generated functional categories were limited by the genes found in the ligand–receptor binding data, which seldom encompassed genes associated with tissue construction and bodily mobility. As a result, the categories exclusively revolved around chemical activities. Nuclear receptors are extensively investigated transcription factors owing to their noteworthy connection with metabolic disorders. Proteins falling under this category can be triggered by the binding of hormones, including sex hormones and insulin, as well as bile acids that play a role in the digestion and absorption of fat-soluble vitamins [51].

Based on our findings, the cluster designated as nuclear receptor displayed six distinct subgroups, each containing a collection of genes associated with abundant target proteins (Figure 4a). The gene that appeared most frequently across various subgroups was the albumin gene (*ALB*), accounting for 108 out of 2159 sentences within the nuclear receptor group. Unlike albumin, sentences originating from different sub-functions of the nuclear receptor were observed to coalesce into distinct subgroups. In the trained dataset, the gene for the vitamin D receptor (*VDR*) was the sole target associated with fat-soluble vitamins, and its corresponding sentences predominantly clustered in cluster 18. Similarly, genes linked to sex hormones, such as androgen (*AR*), estrogen (*ESR*), and progestogen (*PGR*), exhibited a concentration of sentences in cluster 6 (Figure 4b). Importantly, sentences directed towards the peroxisome proliferator-activated receptor (PPAR), strongly linked to adipogenesis, were prominently located in cluster 8. Sentences originating from retinoid X receptor alpha (RXRA) binding as a heterodimer of PPAR [52] were situated in cluster 8 as well (Supplementary Figure S3), suggesting this cluster contains insulin-related binding information (Figure 4b). These results were reproducible using independent, randomly selected data (Supplementary Figure S4).

The model captured characteristics from other functional groups as well. Among the five clusters within the protease group, cluster 19, an independent cluster, emerged with 72.86% of its sentences relating to the human immunodeficiency virus type 1. Of note, HIV protease binding interactions are critical to AIDS antiviral therapy [53]. Interestingly, this cluster featured sentences generated from the renin hormone (REN), following those related to HIV. Given that renin is a dual target for HIV [54], its placement within the cluster further demonstrated the model’s capability to capture biological features.

These observations demonstrate that sentences generated for closely related target proteins are closely located within clusters, suggesting that the sentences generated for multifunctional proteins [55] would be distributed across multiple clusters. We therefore examined sentence distribution across clusters to test this hypothesis. In total, 16 target proteins demonstrated multifunctionality order (Figure 4c), exhibiting instances of singular functionality in cases like RXRA and kinesin-like protein KIF11, as well as spanning across different functional groups, such as PPARD and nitric oxide synthase 3 (NOS3). The presence of PPAR in the protein kinase group stands in line with prior studies [56]. Similar findings included fibroblast growth factor receptor 1 (FGFR1), a TK family member commonly reported to resist tyrosine kinase inhibitors (TKIs) [57]. In our findings, 68.42% of binding interaction sentences from FGFR1 were positioned within the protein kinase group, 26.32% within the nuclear receptor group, and the remaining 5.26% in other groups. Similarly, the renin hormone (RENI) exhibited multifunctional patterns between protein kinases and proteases, and coactivator-associated arginine methyltransferase 1 (CARM1) showed interactions between proteases and phospholipase. However, the catalysis and transferase groups did not showcase distinct multifunctional target proteins.

### 3.4. The Method Highlighted Key Structural Elements Crucial for Drug Discovery

**Figure 5:**
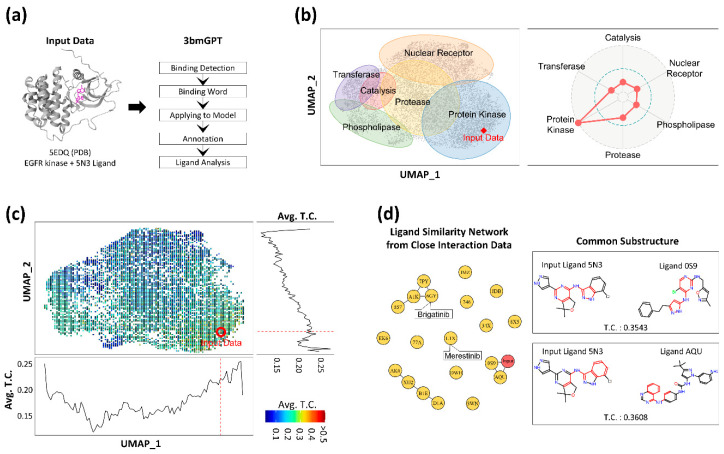
Results of applying 3bmGPT onto new EGFR interaction data. (**a**) Input interaction data and the 3bmGPT analysis process of the input data. (**b**) Biological function analysis. UMAP shows 6 annotated biological functions with approximate areas. A red diamond indicates the location of newly introduced data. The radar chart shows the weights of biological functions calculated for the input data. (**c**) UMAP with binned Tanimoto coefficient (TC) values between the ligand of the input data and the other ligands. Each square with a scaled color indicates the average values of TC values from the included data. Blue color indicates a lower value of TC, while red color indicates higher values. The red circle pinpoints the location of the input data. The bottom line plot shows the TC value pattern according to horizontal UMAP coordinates (UMAP_1). The right line plot shows the TC value pattern according to vertical UMAP coordinates (UMAP_2). The red dashed lines indicate the corresponding position of the input data point. (**d**) Network for ligand similarity with the top 20 closely located data and common structures for connected ligands. In the network plot, circles indicate ligands labeled by PDB IDs. The red node indicates input data, and the line shows similar molecular structures between connected ligands. Common molecular substructures for two connected ligands to the input ligand are illustrated on the right.

To evaluate the 3bmGPT model’s capability in analyzing new data, we introduced new interaction data from the Protein Data Bank (PDB) [27] into the model. We then conducted an in-depth analysis of its binding characteristics, focusing particularly on its ligand component. We utilized the PDB data structure with the ID 5EDQ, representing EGFR kinase, along with its associated ligand (ID 5N3) [58] (Figure 5a). We deliberately excluded the annotations for the PDB, such as information about the target protein and ligand, to assess the model’s performance with unknown data. To elucidate the input’s underlying characteristics, the data were contextualized against the pre-annotated 10,000-sample dataset, which contains biological clusters, ligand information, and known drug associations, to elucidate the input’s latent characteristics. The biological property of the target protein (EGFR) from the input data was accurately determined as a protein kinase through mapping onto a previously annotated UMAP and assessing the prominence of its functional group in a radar plot (Figure 5b). While the data point positioned itself within the protein kinase cluster rather than any other clusters, the radar plot’s depiction of relative weights for each group further indicated that the binding interaction from the input data was closer to the protease cluster than the nuclear receptor cluster. These results are valuable for assessing multifunctionality, especially when the input interaction cannot be unequivocally categorized into a single function.

Moreover, we computed the structural similarity between the ligand from the input PDB and the ligands within the reference datasets. A positive correlation was observed between this structural similarity and proximity in our model’s UMAP space (Figure 5c), suggesting our AI model successfully captures key ligand features. More importantly, the analysis revealed that the interaction-induced features provide a distinct and more holistic grouping compared to clustering based on ligand structure alone. For instance, the protein kinase group, which includes the input EGFR data, was positioned in proximity to the protease and nuclear receptor groups in our interaction space. In contrast, a purely ligand structure-based result showed the kinases closer to the phospholipase group. This distinction is a key finding: it demonstrates that our model’s numerical representation is not solely dependent on ligand structure. Instead, it successfully captures comprehensive binding interaction, underscoring that ligand structure alone is an insufficient descriptor to fully characterize these complex events.

As we discovered the algorithm’s potential to analyze ligands in terms of binding, we attempted to compare the ligands from other closely located binding data with the input PDB’s ligand (ID 5N3). For this, we selected 20 ligand data points closest to the input data. Among these 20 ligands, two (with IDs 0S9 and AQU) displayed molecular structures similar to the input ligand 5N3 (Figure 5d) and shared structural motifs. Notably, two small molecules in the drug database [39], namely merestinib (ID L1X) and brigatinib (ID 6GY), were found in close proximity to the input data. These two drugs are significantly associated with cancer treatment; for instance, merestinib inhibits neurotrophic receptor kinase (NTRK) [59], and brigatinib inhibits anaplastic lymphoma kinase [60]. Although the molecular structures of the two drugs differed from that of 5N3, their inhibition targets were closely related to tyrosine kinase, the same category as the target protein EGFR of the input data.

### 3.5. Benchmarking Highlights the Model’s Capacity for Sensitive Target Detection

**Figure 6:**
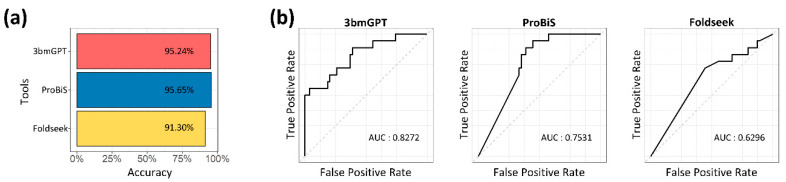
Performance benchmark for target and similar protein identification. (**a**) Bar plot comparing the accuracy of the three tools. The horizontal axis represents the performance accuracy, and the vertical axis lists each of the compared tools. (**b**) Receiver operating characteristic (ROC) curves and corresponding AUC values for the discriminative power in similarity assessment.

We benchmarked our method against ProBiS [40] and Foldseek [41] to evaluate its performance in target protein identification. On a dataset of unlabeled binding data, all three methods successfully identified the correct targets with over 90% accuracy (Figure 6a). While overall detection was comparable, our model’s ability to sensitively distinguish between similar targets, quantified by AUC, was notably superior (Figure 6b). Unlike the other tools, which excel at single-target analysis, our model’s numerical approach to binding characteristics makes it particularly effective for quantitative and comparative assessments.

## 4. Discussion

Transforming 3D chemical data into 1D language-like data has various applications, such as calculating binding affinity [2] and designing novel molecules [17]. New data enriches the model’s “vocabulary” and strengthens its link to pre-trained datasets, improving residue identification and uncovering novel molecular relationships. In our results, key residues from EGFR-TK binding modes indicated possible essential residues for the kinase (Supplementary Figure S1). Adding new bindings to training data can refine the understanding of TK binding characteristics. Clustering quantified binding characteristics as vector values revealed biochemical characteristics consistent with experimental studies. Notably, STK and TK, known for similar ligand binding and biological functions [47,61], showed converging binding traits within the same cluster (Figure 3d), highlighting the LLM’s ability to capture binding attributes numerically. Further molecular insights from a protease cluster (Figure 4a) confirmed prior findings, such as darunavir and aliskiren’s potential dual therapeutic roles [54]. These insights from closely related proteins offer promise for identifying key residues and drug repurposing. The benchmark highlighted the model’s enhanced sensitivity for comparative target evaluation (Figure 6). Thus, protein associations, defined by binding characteristics, are quantifiable via the vector matrix, enabling numerical assessments of interaction and multifunctionality.

Research on ligand–protein complexes suggests the existence of distinct binding pockets for small molecules [62]. AI-driven analyses have since been used to detect and compare macromolecular indentations for pharmaceutical applications [63]. Our study used clustered characteristics to identify ligand interactions in DNA polymerases from different species (Figure 3c). We also identified a nearby cluster of prenyltransferases, grouping them as transferases (Figure 4a), suggesting shared binding pockets or dual-targeting ligands. Notably, farnesyltransferase, a type of prenyltransferase, is linked to DNA synthesis [64,65]. However, its relationship with DNA polymerases remains underexplored. Since DNA polymerase is a key target in drug development [66], leading to agents like actinomycin D [67], mithramycin A [68], and zelpolib [69], understanding its connection to transferases could improve drug discovery.

The use of measurable coordinates facilitated comparisons to identify biological functions proximate to the target protein, thereby unveiling opportunities to uncover potential multifunctionality. This exploration may provide insights into important scaffolds [70] and off-target binding [71]. Drawing from this observation, we demonstrated the potential of this tool in pharmaceutical research (Figure 5d). Specifically, 3bmGPT efficiently positioned an input interaction in close proximity to pre-trained interactions, even with known drugs. This capability enables the tool to effectively search for related drugs, and if drug targets are incorporated into the model, it can be further applied to drug repurposing. As the model was trained with additional drugs and their targets, the representation of simplified binding units as words will be easily archived with minimized data size, connoting progress in the AI model for more accurate output generation.

Apparent limitations inherent to 3bmGPT are as follows: (1) a restricted number of trained target proteins; (2) the presence of uninformative vocabulary vectors stemming from tokenized units; (3) an insufficient number of comparative AI models; and (4) a lack of experimental validation, both for the training dataset and the model’s subsequent predictions. Regarding the first limitation, the training dataset, consisting of a modest 2907 proteins, is relatively limited when compared to the vast number of existing proteins, including their various isoforms. Consequently, substantial room for enhancement exists through the introduction of additional data, thereby enabling the model to scrutinize more intricate protein characteristics. The second limitation pertains to the presence of distinct vectors that fall short in capturing binding interactions comprehensively, as the text-converted binding interactions remain fragmented, aligning with challenges encountered by other LLMs [72]. To enable the reconstruction of tokenized binding modes, further refinement is imperative, potentially involving the development of attention maps to establish connections among the vectors. The third limitation applies to all AI developments as we strive to improve model fit. In this study, only GPT and BERT were compared, but other models could enhance performance. Additionally, variations in parameters, epochs, and tuning could yield different results. Lastly, the model was trained primarily on structural binding data, which lacks quantitative experimental metrics such as binding affinities derived from bioassays. This presents a key limitation, as the correlation between the model’s outputs and actual in vitro binding strength remains unconfirmed, leaving its practical application potential in question. Although 3bmGPT’s results show a favorable alignment with established findings, its capacity to discover novel ligands still necessitates rigorous prospective validation under laboratory conditions. Consequently, further research is essential to enhance and validate the model’s performance. Promising future direction includes (1) fine-tuning the model using feedback from a true/false binding classification task and (2) employing knowledge distillation to create smaller, specialized models tailored to the unique binding characteristics of specific protein families.

## 5. Conclusions

In this study, we developed a robust method and implemented it in a novel language model, 3bmGPT. Our results demonstrate that 3bmGPT can effectively predict key residues for proteins with similar biological functions (Figure 3) and successfully convert 3D binding modes into text to classify molecular characteristics (Figure 4). Furthermore, the model identified meaningful connections between biological functions via biochemical interactions (Figure 5). Its superior performance in sensitive target detection was validated through benchmarking (Figure 6). To facilitate further research and ensure reproducibility, the pre-trained model and all associated codes for training and analysis are openly available.

## Figures and Tables

**Table 1:** Training times by WSE.

	GPT-2 (Small)		GPT-2 (Medium)	
	Word-Based	BPE-Based	Word-Based	BPE-Based
Training time	2 h	1 h	3 h	2 h

## Data Availability

The raw data utilized in this study were sourced from the Crossdocked2020 dataset [18], which is publicly available at https://github.com/gnina/models (accessed on 1 April 2023). The pre-trained model and associated analysis data generated during this study are available on Zenodo (https://doi.org/10.5281/zenodo.17053576 (accessed on 4 September 2025)). The open-source code used for training and analysis can be accessed at https://github.com/LeeTS1001/3bmGPT (accessed on 1 September 2025).

## Supplementary Materials

The following supporting information can be downloaded at https://www.mdpi.com/article/10.3390/molecules30234516/s1, Figure S1: Binding interaction words generation for EGFR by 3bmGPT; Figure S2: Plotting target proteins on UMAP coordinates. Red dots represent interaction data points for each target protein; Figure S3: Density plot on UMAP coordinates for sentences from PPARG, PPARD, and RXRA; Figure S4: Reproduced result of density plot on UMAP coordinates; Table S1: Majority proportion of sentences per target protein in matched cluster.
