# Supplementary material for: Systematic Exploration of Small-Molecule Binding via a Large Language Model Trained on Textualized Protein–Ligand Interactions
Source: Molecules. 2025 Nov 22;30(23):4516. doi: 10.3390/molecules30234516 (PMC12692874; doi:10.3390/molecules30234516)
Supplement: Supplementary file 1 [file molecules-30-04516-s001.zip › molecules-3979952-supplementary.pdf]

Supplementary Materials

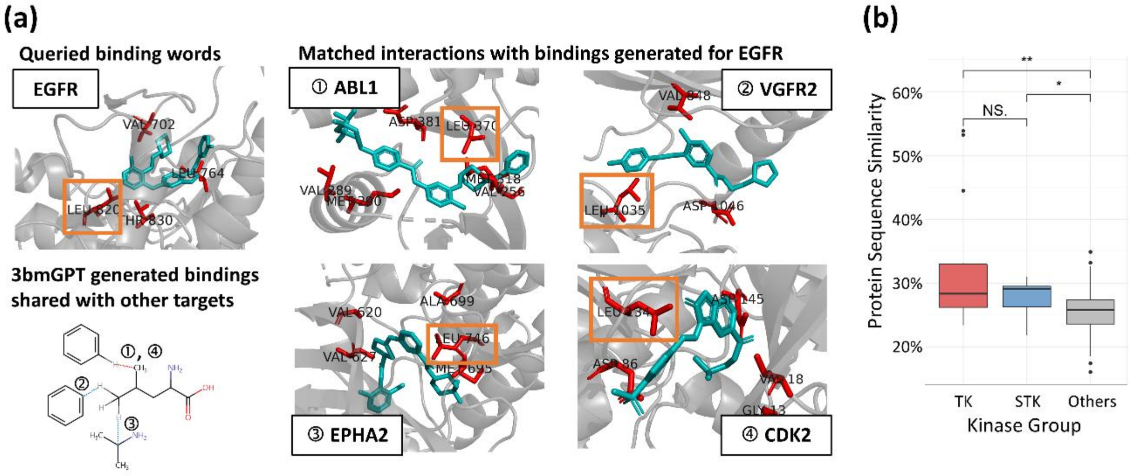

Supplementary Figure S1. Binding interaction words generation for EGFR by 3bmGPT. (**a**) 3D ligand binding structure for generated binding interaction words of EGFR and matched words of other target proteins; ABL1, VGFR2, EPHA2, and CDK2. Blue color represents ligand and red color represents amino acid from pocket of target protein. Orange boxes emphasize matched binding interactions between EGFR and the others. (**b**) kinase group boxplots for protein sequence similarities with EGFR. Red boxplot is from the proteins related to tyrosine kinase (TK), blue boxplot is from proteins related to serine/threonine kinase (STK), and grey boxplot is from the other proteins. Statistical significance for difference between them were measured by t-test: **$p < 0.01$; *$p<0.05$; NS, not significant.

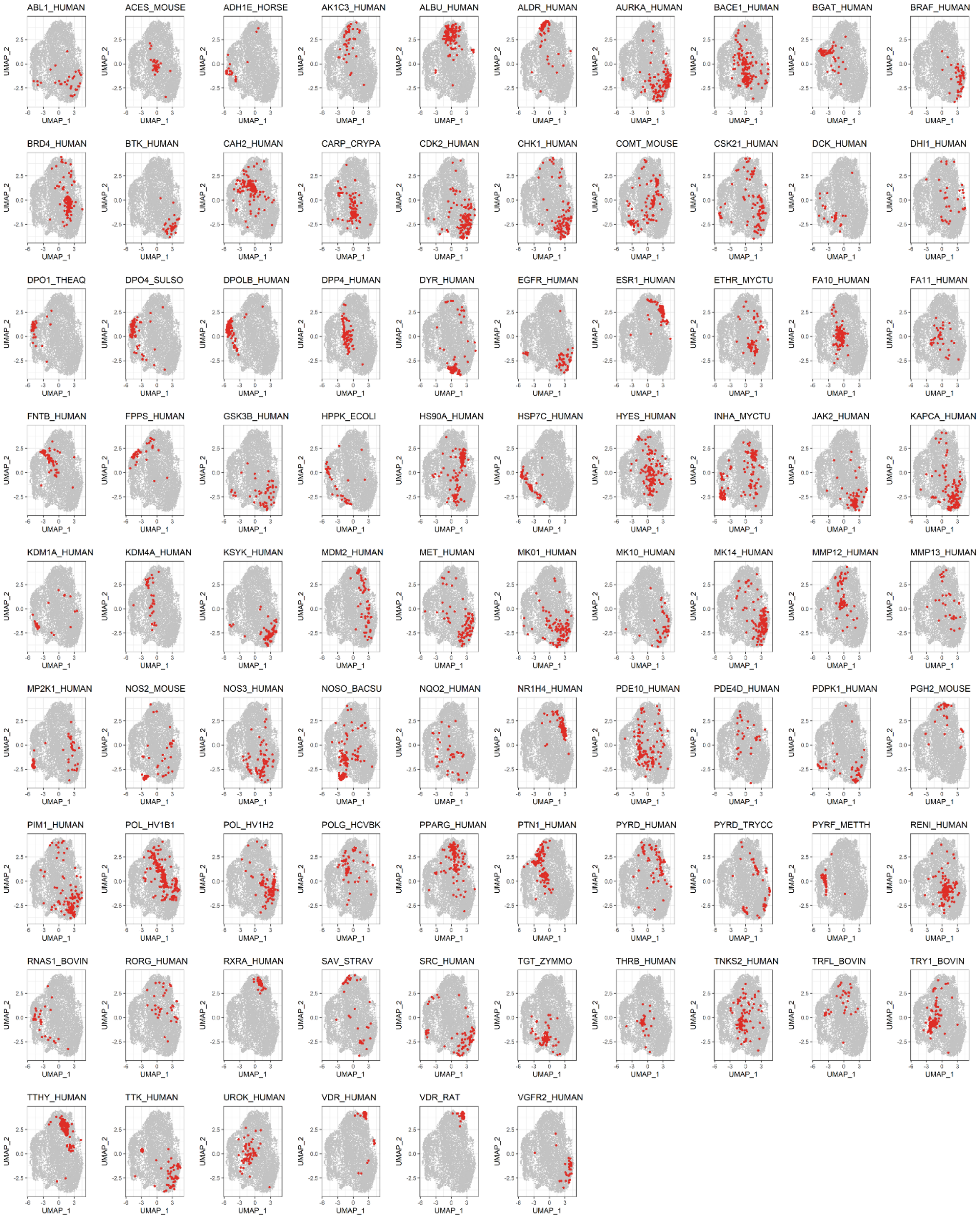

Supplementary Figure S2. Plotting target proteins on UMAP coordinates. Red dots represent interaction data points for each target protein.

Target	BTK_HUMAN	IRAK4_HUMAN	JAK2_HUMAN	KAPCA_HUMAN	CAH2_HUMAN	FA11_HUMAN	FA7_HUMAN	THRB_HUMAN	TRY1_BOVIN
Cluster	0	0	0	0	1	1	1	1	1
Proportion	59%	76%	68%	51%	63%	66%	62%	65%	66%

Target	UROK_HUMAN	ACES_MOUSE	ACES_TETCF	FA10_HUMAN	PTN1_HUMAN	BRAF_HUMAN	EPHA2_HUMAN	VGFR2_HUMAN	DPP4_HUMAN
Cluster	1	2	2	2	2	3	3	3	4
Proportion	71%	88%	63%	60%	51%	61%	59%	69%	66%

Target	ADH1E_HORSE	CARM1_HUMAN	KDM1A_HUMAN	ANDR_HUMAN	ESR1_HUMAN	ESR2_HUMAN	DPO1_THEAQ	DPO4_SULSO	DPOLB_HUMAN
Cluster	5	5	5	6	6	6	7	7	7
Proportion	85%	69%	69%	78%	83%	88%	75%	78%	87%

Target	DPOLL_HUMAN	POLH_HUMAN	TRPD_MYCTU	ALBU_HUMAN	PPARD_HUMAN	RXRA_HUMAN	POL_HV1H2	ALDR_HUMAN	FOLH1_HUMAN
Cluster	7	7	7	8	8	8	9	10	10
Proportion	92%	90%	63%	59%	52%	91%	51%	71%	58%

Target	CARP_CRYPA	RENI_HUMAN	TGT_ZYMMO	NR1H4_HUMAN	DYR_HUMAN	DYR_STAAU	HS90A_HUMAN	BAZ2B_HUMAN	BRD4_HUMAN
Cluster	11	11	11	12	13	13	13	14	14
Proportion	76%	53%	78%	57%	66%	56%	63%	64%	76%

Target	TTHY_HUMAN	PYRF_METTH	VDR_HUMAN	VDR_RAT
Cluster	15	16	18	18
Proportion	84%	82%	82%	85%

Supplementary Table S1. Majority proportion of sentences per target proteins in matched cluster.

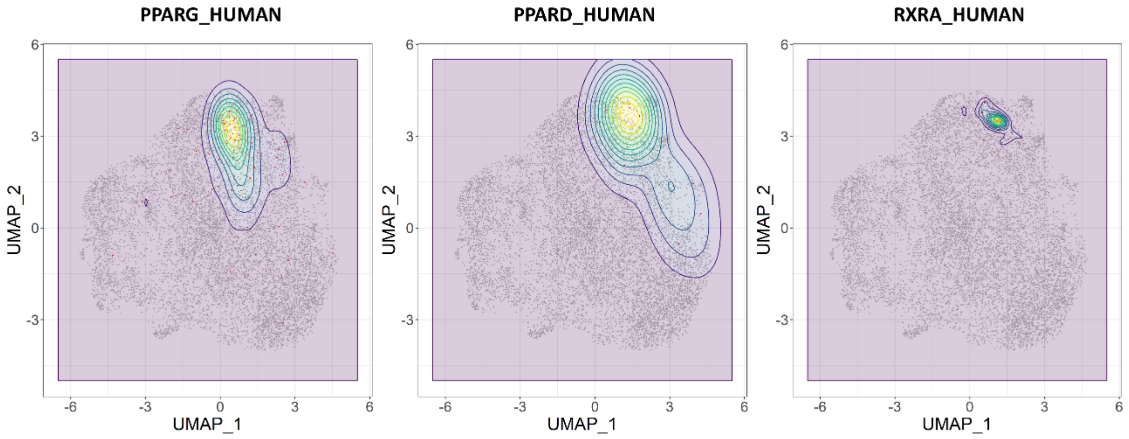

Supplementary Figure S3. Density plot on UMAP coordinates for sentences from PPARG, PPARD, and RXRA.

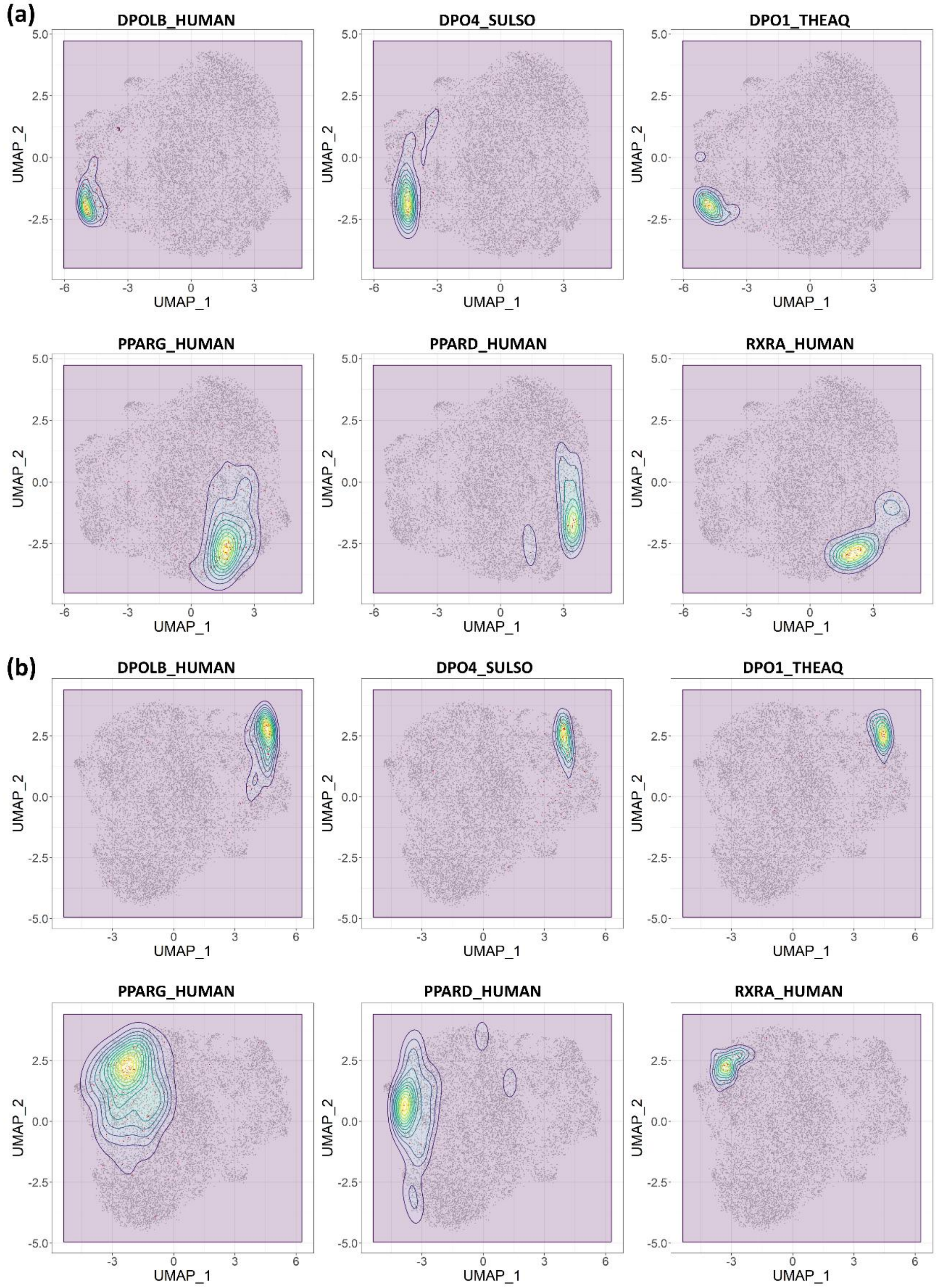

Supplementary Figure S4. Reproduced result of density plot on UMAP coordinates. (**a**) 1st reproduced result of density plots for DNA polymerase and insulin related targets (**b**) 2nd reproduced result of density plots for DNA polymerase and insulin related targets
